# Preclinical antitumor activity of SST0116CL1: A novel heat shock protein 90 inhibitor

**DOI:** 10.3892/ijo.2014.2575

**Published:** 2014-08-01

**Authors:** LOREDANA VESCI, FERDINANDO MARIA MILAZZO, VALERIA CAROLO, SILVIA PACE, GIUSEPE GIANINI

**Affiliations:** Research & Development, Sigma-Tau Industrie Farmaceutiche Riunite S.p.A., Pomezia, Italy

**Keywords:** heat shock protein 90, antitumor, client proteins

## Abstract

4-Amino substituted resorcino-isoxazole (SST0116CL1) (property of Sigma-Tau Research Switzerland S.A.) is a potent, second generation, small-molecule heat shock protein 90 inhibitor (Hsp90i). SST0116CL1 binds to the ATP binding pocket of Hsp90, and interferes with Hsp90 chaperone function thus resulting in client protein degradation and tumor growth inhibition. The aim of the study was to assess SST0116CL1 in various solid and haematological tumors. The antitumor properties of SST0116CL1 were assessed using *in vitro* cell proliferation and client protein degradation assays and *in vivo* different tumor xenograft models. Pharmacokinetic (PK) data were also generated in tumor-bearing mice to gain an understanding of optimal dosing schedules and regimens. SST0116CL1 was shown to inhibit recombinant Hsp90α and to induce the destabilization of different client proteins, often overexpressed and constitutively activated in different types of hematological or solid human tumors. In preclinical *in vivo* studies, it was revealed to induce antitumor effects in murine models of leukemia and of gastric and ovarian carcinoma. A modulation of PD biomarkers in terms of downregulation of Hsp90 client proteins in tumor-bearing mice was found. SST0116CL1 is a new clinical candidate for cancer therapy. The antitumor property of SST0116CL1, likely due to direct inhibition of the Hsp90 enzymatic activity, may prove to be a critical attribute as the compound enters phase I clinical trials.

## Introduction

Heat shock protein 90 (Hsp90) is a molecular chaperone that contributes to the maintenance of the correct folding of critical protein effectors involved in cell survival, growth and differentiation (1). Hsp90, in association with other cochaperone proteins, catalyzes via its ATPase activity the conformational changes of client proteins, that are mainly involved in cell signalling, proliferation and survival (2). In addition, Hsp90 ensures the stability of a number of mutated client proteins required for tumor growth and resistance (3). In recent years this molecular target has gained considerable interest for the discovery and development of novel anticancer drugs (4,5), because of their putative therapeutic use in multiple cancer indications.

Thus, a number of novel synthetic Hsp90 inhibitors, such as NVP-AUY922 (Novartis/Vernalis) (6), STA-9090 (Synta) (7), AT13387 (Astex) delivered intravenously and XL-888 (Exelixis), DS-2248 and Debio 0932 orally administered (8), are currently under oncology clinical investigations. Besides having the unusual ability of disrupting the activity of many receptors, kinases and transcription factors, all these drugs overcome the potential limitations of the ansamycin-derived Hsp90 inhibitors including complex formulations, poor solubility and hepatotoxicity (8).

Preclinical data in human tumor xenograft models show that these Hsp90 inhibitors are efficacious in a wide variety of tumor types, through activity against different oncoproteins (9). Antitumor efficacy ranges from minimal effects to tumor growth stasis, but rarely leads to tumor regression (10–15). The different efficacy of antitumor response in various xenograft models may be attributable to differences in client protein dependence on Hsp90, tumor dependence on the client protein, kinetics of client protein degradation and turnover, as well as drug pharmacokinetic and pharmacologic properties. This complexity makes it difficult to predict antitumor response in xenograft models and renders patient stratification in the clinic challenging (3). Since Hsp90 also plays a key role in regulating protein function and stability in normal cells (16), the balancing of efficacy and toxicity is important to achieve as suitable therapeutic index in patients.

We have previously described the discovery of a novel series of 3,4-isoxazolediamides based Hsp90 inhibitors which were shown to have a very interesting activity both *in vitro* and *in vivo* (17). In particular, within this group, we selected the compound SST0116CL1 as a synthetic, new chemical entity designed to potently inhibit Hsp90. SST0116CL1 binds to the ATP binding pocket of Hsp90, and interferes with Hsp90 chaperone function thus resulting in client protein degradation and tumor growth inhibition.

We report on the *in vitro* activity and *in vivo* pharmacokinetic and efficacy profiles of SST0116CL1 in human cancer cell lines from different etiology. These results support the selection of SST0116CL1 for clinical development.

## Materials and methods

### Compound preparation

**Figure 1:**
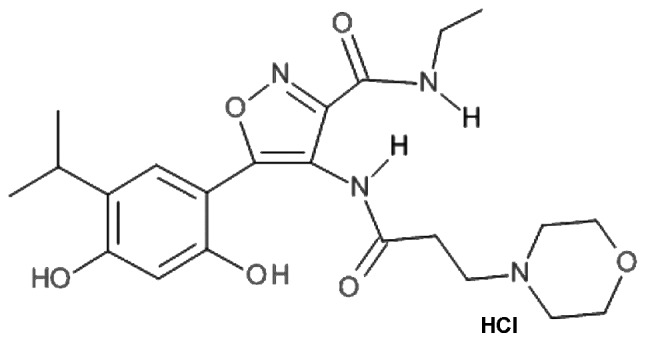
Chemical structure of SST0116CL1.

For *in vitro* experiments, stock solutions of SST0116CL1 (property of Sigma-Tau Research Switzerland S.A) (see Fig. 1) were prepared in 100% dimethyl sulfoxide (DMSO) at 10 mM and stored at −20°C. For intraperitoneal or intravenous administration, SST0116CL1 was formulated in 2.5% ethanol, 20% 50 mM tartaric acid, 77.5% (5% glucose in water containing 1% Tween-80) vol/vol and delivered in a volume of 10 ml/kg.

### Binding on Hsp90 by a fluorescence polarization assay

GM-FITC, supplied by Invivogen (cat. no. 06C23-MT, San Diego, CA, USA), was previously dissolved in DMSO to obtain 10 mM stock solutions and kept at −20°C until use. Recombinant human Hsp90, purchased from Stressgen (cat. no. SPP-776, Victoria, BC, Canada), was previously dissolved in assay buffer (HFB) to form 2.2 μM stock solutions and kept at −80°C until use. On the day of the experiment, compound solutions at various concentrations were prepared by serial dilutions in assay buffer (HFB) containing 20 mM HEPES (K^+^), pH 7.3, 50 mM KCl, 5 mM MgCl_2_, 20 mM Na_2_MoO_4_, and 0.01% NP-40. Before each use, 0.1 mg/ml bovine γ-globulin and 2 mM DTT were added. Fluorescence polarization (FP) was performed in Opti-Plate-96F well plates (Perkin-Elmer, Zaventem, Belgium) using the Wallac Envision 2101 multilabel plate reader (Perkin-Elmer). To evaluate the binding affinity of the molecule, 50 μl of the GM-FTC solution (5 nM) were added to 30 nM Hsp90 in the presence of 5 μl of the test compounds at increasing concentrations. The plates were shaken at 4°C for 4 h, and the FP values in mP (millipolarization units) were then recorded. The IC_50_ value was calculated as the inhibitor concentration that displaced 50% of the tracer, each data point being the result of the average of triplicate wells, and was determined from a plot using non-linear least-squares analysis. Curve fitting was performed using Prism GraphPad software program (GraphPad Software, Inc., San Diego, CA, USA).

### Cell lines and cell sensitivity to drug

A non-small cell lung carcinoma (NSCLC, NCI-H460) cell line, a breast carcinoma (BT-474) cell line, a fibrosarcoma (HT-1080) cell line and an acute monocytic leukemia (MV4;11) cell line were purchased from the American Type Culture Collection (Manassas, VA, USA). The sensitive ovarian carcinoma cell line (A2780) was from European Collection of Animal Cell Cultures (ECACC). The gastric carcinoma (GTL-16) and the epidermoid carcinoma (A431) cell lines were kindly provided by Metheresis and by Istituto Tumori di Milano, respectively. The NSCLC, the breast carcinoma and the epidermoid carcinoma cells, as well as the acute monocytic leukaemia and the ovarian carcinoma cells were grown in RPMI-1640 (Lonza, Verviers, Belgium) supplemented with 10% fetal bovine serum (FBS, Invitrogen, Geithersburg, MD, USA). The gastric carcinoma GTL-16 cells were grown in DMEM (Lonza) supplemented with 10% fetal bovine serum (FBS, Invitrogen). The fibrosarcoma cells were grown in EMEM (Lonza) supplemented with 10% fetal bovine serum (FBS, Invitrogen). Cells were routinely maintained in a humidified atmosphere with 5% CO_2_ at 37°C. All experiments were performed starting from frozen cell stocks of each cell line. When thawed, such cells were characterized in house, by assessing cell morphology, cell growth kinetics curve and absence of mycoplasma. The cell sensitivity to the drug was measured *in vitro* by assessing the inhibition of proliferation by sulphorodamine B (SRB) assay. Briefly, cells were seeded in 96-well tissue culture plates in complete medium (10% FBS), and 24 h after seeding were treated for 72 h with various concentrations of SST0116CL1. The drug cytotoxic potency was evaluated by means of the ‘ALLFIT’ computer program and defined as IC_50_ (drug concentration required for 50% inhibition of cell survival).

### Her2 degradation assay

BT-474 cells were seeded into Viewplates-384TC (cat. 6007480, Perkin-Elmer Inc., Waltham, MA, USA) at the density of 2×10^4^ cells/well in 100 μl of culture medium, and were then incubated at 37°C, in the presence of 5% CO_2_ for 24 h. Different concentrations of the drug or vehicle (DMSO) were then added to each well, and cells were cultured for further 24 h. After washing the cells with PBS, 30 μl of Her2 AlphaLISA Immunoassay buffer were added to wells and mixed on a shaker at 22°C for 30 min. Then 15 μl of a solution (10 μg/ml) consisting in Her2 AlphaELISA Anti-ERBB2/Her2 Acceptor beads and (1 nM) Biotinylated Anti-ERBB2/Her2 Antibody were added to wells, and incubated at 22°C for 2 h. Finally, 5 μl of (40 μg/ml) Streptavidin Donor beads were added, and the plate was incubated at 22°C for 1 h, in the dark, and measurements were done through the multilabel reader Envision (Wallac Envision 2101 multilabel reader, Perkin-Elmer, Zaventem, Belgium). To rule out that the declining signal in drug-treated cells was not the result of reduced cell number caused by unspecific cell death but due to decreased Her2 content, suitable viability studies were performed by means of the luminescence ATP detection assay system (ATPlite 1 Step cat. 6016941, Perkin-Elmer, Zaventem, Belgium). IC_50_ was calculated as the drug concentration needed to degrade 50% of the total Her2; each data point was the result of the average of triplicate wells, and determined from a plot using nonlinear least-squares analysis. Curve fitting was performed using the Prism GraphPad software program (GraphPad software, Inc., San Diego, CA, USA).

### Western blot analysis

For the *in vitro* experiments, A431 (human epidermoid carcinoma) cells were seeded at 1×10^6^ cells/100 mm dish in complete culture medium, and allowed to grow overnight at 37°C with 95% air and 5% CO_2_. The day after, the medium was renewed and cells were treated, for 24 h, with various concentrations of SST0116CL1. 17-DMAG (at the concentration of 0.2 μM) was used as internal reference inhibitor. Following treatments, cells were rinsed twice with ice-cold PBS and then lysed in RIPA buffer supplemented with protease and phosphatase inhibitors. After determination of the protein concentration by Bradford Protein Assay (Thermo Scientific, Rockford, IL, USA), equal amounts of cellular extracts were separated by SDS-PAGE and then transferred onto nitrocellulose membranes. Non-specific binding sites were blocked by incubation of the membranes with 5% non-fat dry milk in TBS, overnight at 4°C. Membranes were finally probed with the following primary antibodies: anti-EGFR (Upstate Biotechnology, Millipore Corporate, Billerica, MA, USA); anti-Cdk4 (Santa Cruz Biotechnology Inc., Santa Cruz, CA, USA); anti-Akt (Cell Signaling Technology, Inc., MA, USA); anti-HSP70 (BRM-22) and anti-Actin (Sigma Chemical Co., St. Louis, MO, USA). After extensive washings in TBS, immunoreactive bands were revealed by horseradish peroxidase-conjugated secondary antibodies, using an enhanced chemiluminescence detection reagent (ECL Plus, GE Healthcare Bio-Sciences, Uppsala, Sweden), and acquired by a phosphoimaging system (STORM 860; Molecular Dynamics, Sunnyvale, CA, USA). Protein loading equivalence was corrected in relation to the expression of actin. For quantification of signals, blots were subjected to densitometry analysis.

### Murine xenograft model

All experiments were carried out at Sigma-Tau (Rome, Italy) using 5–6 week-old female athymic nude mice (Harlan, Italy). Mice were maintained in laminar flow rooms with constant temperature and humidity. Experimental protocols were approved by the Ethics Committee for Animal Experimentation of Sigma-Tau according to the United Kingdom Coordinating Committee on Cancer Research Guidelines. The following human tumor xenograft models were used for antitumor activity studies: GTL-16 gastric carcinoma, MV4;11 AML, A2780/ADR multi-drug resistant ovarian carcinoma. Exponentially growing tumor cells (5×10^6^/mouse) were s.c. inoculated in the right flank of nude mice. Groups of eight mice were employed to assess antitumor activity. Drug treatments were started 3 or 4 days after tumor injection. SST0116CL1 was delivered intraperitoneally or intravenously in a volume of 10 ml/kg according to different schedules (qdx5/w: daily from Monday to Friday; q2d/w: Monday, Wednesday, Friday; q4d/w: Monday and Friday). Tumor growth was followed by measurement of tumor diameters with a Vernier caliper. Tumor volume (TV) was calculated using the formula: TV (mm^3^) = [d^2^ × D]/2, where d and D are the shortest and the longest diameter, respectively. The efficacy of the drug treatment was assessed as: TV inhibition percentage (TVI%) in treated vs. control mice, calculated as: TVI% = 100 − [(mean TV treated/mean TV control) ×100)]. CR (complete response) was defined as no evidence of tumor at the end of the drug-treatment. When tumors reached a maximum volume of 2,000 mm^3^, mice were sacrificed by cervical dislocation. To examine the possible toxicity of treatment, body weight was recorded throughout the study. BWL% (body weight loss) was calculated as 100 − [(mean BW_dayx_/mean BW_day1_)] ×100), where day 1 is the first day of treatment and day × is any day after (maximum BWL%). In order to assess the *in vivo* effect of SST0116CL1 on the expression of typical HSP90 client proteins, GTL-16 tumor xenografts (4 samples/group) were excised at different times after the last treatment, and then total protein lysates were prepared through the homogenization of tumor samples in T-PER (Tissue Protein Extraction Reagent, Pierce, Rockland, IL, USA), supplemented with 10 μg/ml of protease inhibitor cocktail (Sigma Chemical Co., St. Louis, MO, USA). Determination of the protein concentration and western blot analysis were finally performed as previously described for the *in vitro* experiments.

### PK sampling and analysis

CD1 nude mice bearing A431 epidermoid carcinoma xenografts were used. Mice were treated with a single intraperitoneal dose of SST0116CL1 at 80 mg/10 ml/kg. Levels of SST0116CL1 were determined in blood, lung and tumor samples collected at 1, 2, 4, 8 and 24 h post-treatment. Bioanalysis was conducted by quantitative HPLC-MS/MS and PK analysis was carried out according to a non-compartmental approach for sparse data sampling (WinNonLin, Pharsight). The maximum plasma concentration (C_max_) of SST0116, and the corresponding times of occurrence (T_max_) were obtained directly from the mean plasma or tissue concentration data. The slope of the terminal disposition phase (k) was determined by logarithmic-linear regression and used to calculate the terminal half-life (T_1/2_) as 0.693/k. The linear trapezoidal rule was used to estimate the area under the plasma concentration versus time curve from zero to the last time point with a measurable drug concentration (C_last_) for AUC_0-last_, and extrapolating to infinity by addition of the quantity C_last_/k for AUC_0-inf_. The apparent total clearance, (CL/F) and the apparent terminal volume of distribution (Vz/F) referenced to plasma were calculated as Dose/AUC_0-inf_, and Dose/(k*AUC_0-inf_), respectively.

### Statistical analysis

The data shown represent mean values ± standard error of mean (SEM) and standard deviation (SD). For comparison between a control and a treatment group, an unpaired Mann-Whitney test was used. A P-value ≤0.05 was considered significant.

## Results

### SST0116CL1 inhibits cell growth, and recombinant HSP90α

In a competitive binding fluorescence polarization assay, SST0116CL1 was shown to inhibit recombinant Hsp90α, with an IC_50_ value of 0.2 μM (Table I). The ability of SST0116CL1 to induce degradation of Her2 in BT-474 human breast carcinoma cells, was also measured by means of a specific Her2 AlphaELISA immunoassay. As shown in Table I, exposure of BT-474 cells for 24 h to increasing concentrations of SST0116CL1 was able to cause a significant reduction in cellular levels of Her2, with an IC_50_ value of 0.2 μM. The ability of SST0116CL1 to target Hsp90 directly on human tumor cells was evaluated by a tumor cell proliferation assay against a representative panel of human tumor cell lines, including fibrosarcoma (HT-1080), acute monocytic leukemia (MV4;11), NSCLC (NCI-H460), ovarian (A2780), epidermoid (A431), gastric (GTL-16), breast (BT-474) carcinoma cell lines, characterized by constitutively activated oncogenic pathways (Table II). SST0116CL1 was effective in inhibiting cell growth in all the different tumor cell lines, following 72-h exposure, with IC_50_ values ranging from 0.1 to 0.8 μM (Table II). Because the broad mechanism of action of SST0116CL1, such as for other Hsp90 inhibitors, it was not possible to associate the sensitivity observed in the cell panel with the mutation of a single gene. These results correlated well with those obtained from the Her2 degradation assay as well as with the Hsp90 competitive binding assay.

### SST0116CL1 inhibits the expression of specific Hsp90 client proteins

**Figure 2:**
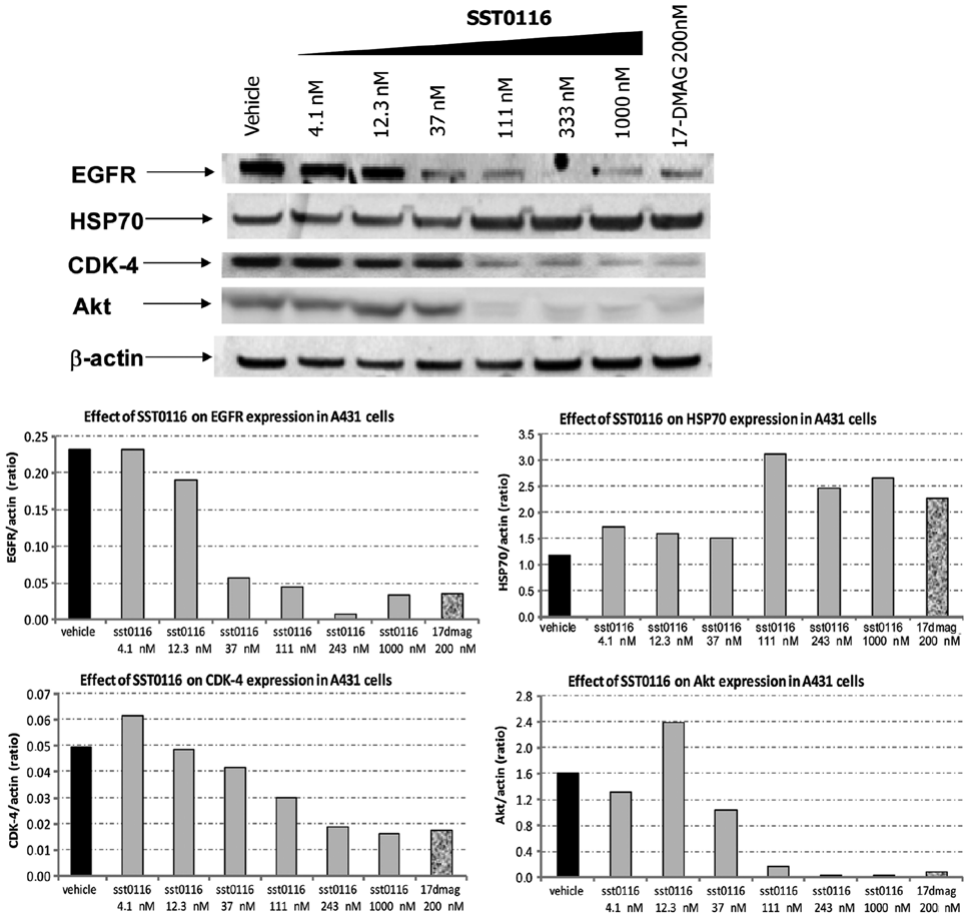
SST0116CL1 downregulates Hsp90 client protein levels (EGFR, Akt, CDK-4) and upregulates Hsp70 in A431 epidermoid carcinoma cells. Total cellular extracts were obtained 24 h after treatment. 17-DMAG was used as positive internal reference. Actin is shown as a control for protein loading. A representative blot is shown. Results of densitometry analysis were reported as normalized (to actin) ratios. Western blot experiments were performed at least twice resulting in very similar results.

The ability of SST0116CL1 to downregulate the expression of a few representative Hsp90 protein clients and to induce the expression of Hsp70 protein was assessed, by western blot analysis, in the A431 human squamous carcinoma cell line, characterized by the constitutive overexpression of EGFR. As shown in Fig. 2, a dramatic depletion of selected Hsp90 client proteins (EGFR, CDK4 and AKT), associated to a very strong increase in the expression levels of Hsp70, was achieved in A431 cells after a 24-h exposure of cells even to concentrations of SST0116 almost 10-fold lower than the cytotoxic doses of the drug. Thus, the modulation of client proteins and the upregulation of Hsp70 cochaperone were consistent with inhibition of Hsp90 function, and confirmed that target modulation of Hsp90 was achieved.

### SST0116CL1 inhibits tumor growth in different cancer cell xenografts

**Figure 3:**
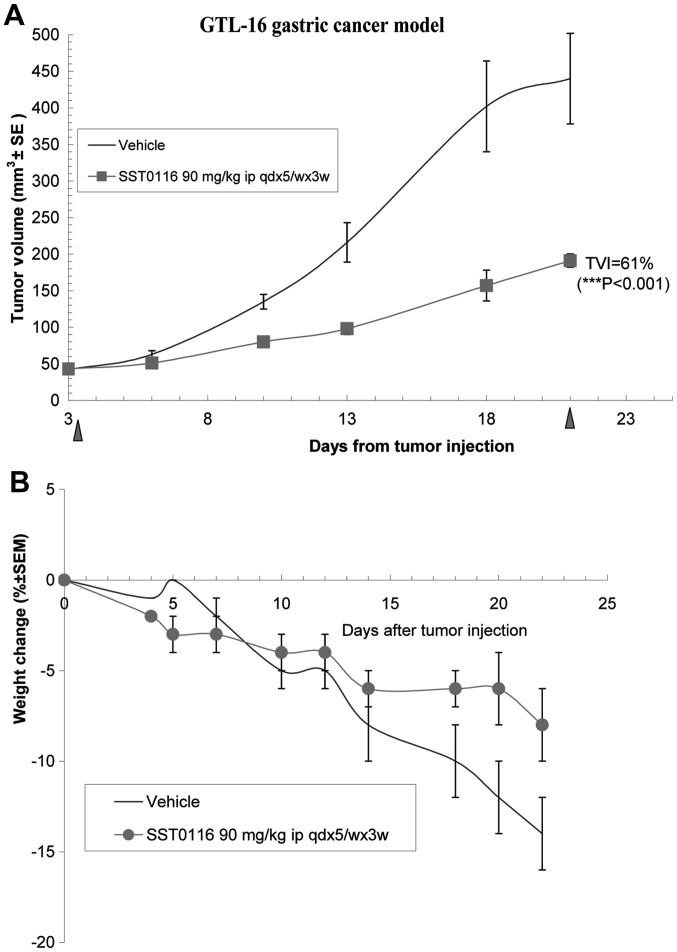

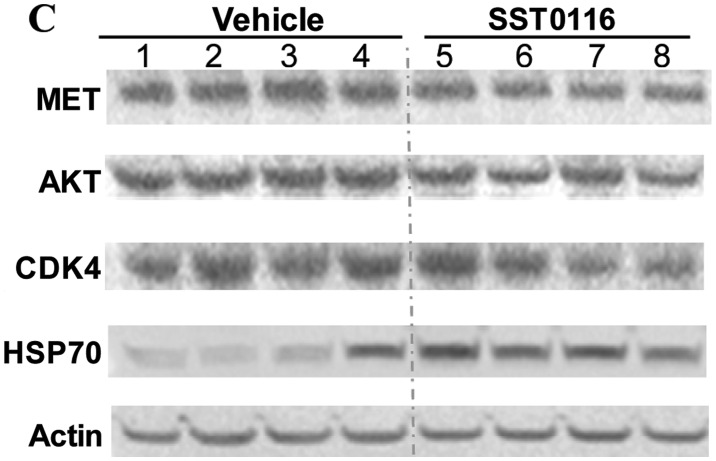
SST0116CL1 (90 mg/kg, i.p.; qdx5/wx3w) (A) significantly inhibits tumor progression in gastric cancer model (GTL-16) and (B) protects mice from cachexia induced by the tumor. Treatment started when tumors reached 50 mm^3^ (^***^P<0.001 vs. vehicle control; Mann-Whitney test; n=8/group). (C) SST0116CL1 (lanes 5–8) downregulates Hsp90 client protein levels (c-MET, AKT, CDK4) and upregulates Hsp70, with respect to vehicle-treated mice (lanes 1–4), in GTL-16 tumor samples collected 6 h after the last treatment. Actin is shown as a control for protein loading. Representative blots of four tumor samples/group are shown.

**Figure 4:**
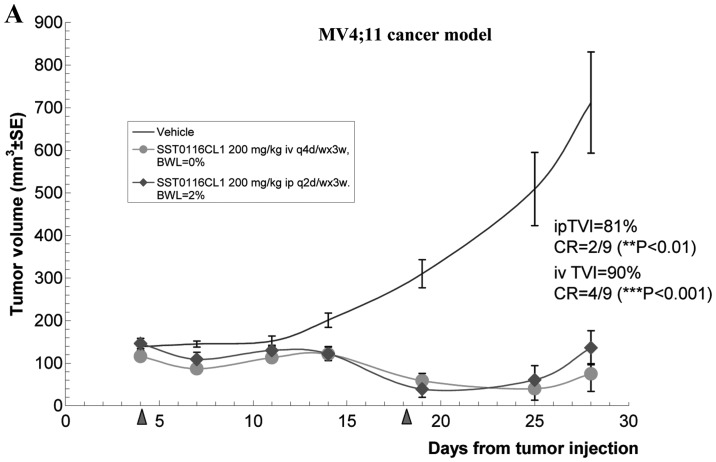

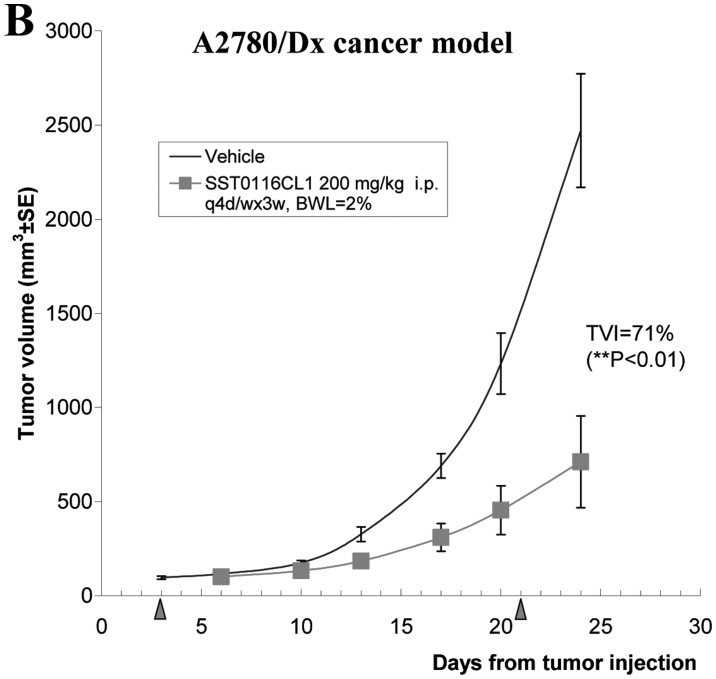
SST0116CL1 significantly inhibits (A) tumor progression in AML (MV4;11) and (B) multidrug-resistant ovarian carcinoma (A2780/Dx). Body weight loss (BWL%) was also reported. Treatment started when tumors reached 50–100 mm^3^. [^**^P<0.01 and ^***^P<0.001 vs. vehicle control (Mann-Whitney test, n=8 per group].

SST0116CL1 significantly reduced solid tumor growth in different xenograft models. Against the gastric carcinoma (GTL-16), SST0116CL1, administered at 90 mg/10 ml/kg i.p. according to the schedule qdx5/wx3w, was able to induce a significantly Tumor Volume Inhibition of 61% (P<0.001; Fig. 3A). Interestingly, the compound revealed to be well-tolerated and to protect mice from cachexia induced by this tumor xenograft (see the body weight of treated group in comparison with the drug-treated group) (Fig. 3B). To validate that the *in vivo* antitumor effect of SST0116CL1 was effectively related to the inhibition of Hsp90, the modulation of selected Hsp90 client proteins was assessed by western blot in tumor xenografts a few hours after the last treatment. As shown in Fig. 3C, SST0116CL1 induced a relevant decrease of the protein levels of three typical client proteins (c-MET, AKT and CDK4) in GTL-16 tumor lysates and, at the same time, significantly increased the expression levels of the chaperone Hsp70, thus confirming that inhibition of Hsp90 function was achieved. The *in vivo* antitumor efficacy of SST0116CL1 against MV4;11 AML cell line was also investigated. Administration of SST0116CL1 (200 mg/10 ml/kg) intravenously (q4d/wx2w) and (200 mg/10 ml/kg) intraperitoneally (q2d/wx2w) showed a very potent antitumor effect (TVI=90%, P<0.001, with 50% of complete response, and TVI=81%, P<0.01, with 22% of complete response, respectively) (Fig. 4A). Thus, the antitumor efficacy was comparable irrespectively of the routes and schedules. In both treatments, SST0116CL1 was revealed to be well tolerated. The A2780/Dx ovarian tumor cell line, overexpressing P-glycoprotein, also showed a significant responsiveness to the compound (TVI=71%, P<0.01). Against this tumor model, SST0116CL1 was delivered at 200 mg/10 ml/kg i.p. according to the schedule q4d/wx3w, and again it did not show any toxicity (Fig. 4B).

### SST0116CL1 shows a preferential distribution in tumors

**Figure 5:**
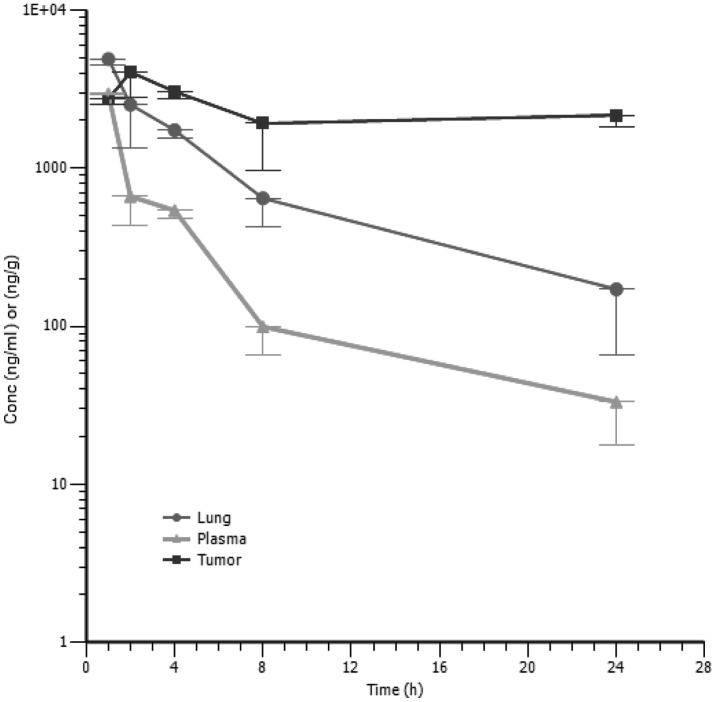
Plasma and tissue concentration vs. time profiles for SST0116CL1 administered as a single intraperitoneal dose of 80 mg/kg in tumor-bearing mice (A431 epidermoid carcinoma).

The PK profile of SST0116CL1 in tumor bearing animals was determined after a single dose of 80 mg/10 ml/kg of compound administered intraperitoneally. The concentrations of SST0116CL1 were assessed in both blood and tissue samples (tumor and lung). A C_max_ of 2,953 ng/ml was observed in plasma 1 h after dosing, then plasma concentrations decreased according to a bi-exponential profile, having a terminal half-life of 5.8 h. As suggested by its plasma concentration versus time profile, SST0116CL1 distributed outside the systemic circulation, reaching C_max_ of 4,890 ng/ml in lung and 4,046 ng/ml in tumor within 1 and 2 h, respectively (Table III). The terminal elimination phase in lung paralleled that of plasma (T_1/2_ of 6.5 h); conversely SST0116CL1 seemed to accumulate in tumor; in fact, in this tissue its concentration declined much more slowly than in plasma and lung (Fig. 5). The tissue to plasma AUC_0-last_ ratios turned out to be about 3 for lung and 8 for tumor; thus these data reveal enhanced tissue distribution. The CL/F and Vz/F evaluated from plasma data were 11,268 ml/h/kg and 94,551 ml/kg, respectively. The high values of CL/F and Vz/F might also reflect an incomplete bioavailability of the tested compound in nude mouse.

## Discussion

Hsp90 is a component of a molecular chaperone complex that plays critical roles in regulating the folding, maturation and stabilisation of key signalling molecules which control cell proliferation, survival and transformation. Hsp90 works in association with other cochaperones and catalyzes, via its ATPase, the conformational changes of a set of cancer-associated proteins, collectively referred to as ‘clients’. Inhibition of Hsp90 causes simultaneous destabilization and eventual degradation of client proteins that in turn result in suppression of tumor growth. A number of novel synthetic Hsp90 inhibitors are currently under oncology clinical investigations for the treatment of a wide variety of tumor types (8,18,19). The earlier geldanamycin analogues (i.e., 17-AAG or 17-DMAG), despite potent *in vitro* and *in vivo* preclinical activity, have not shown clear clinical benefit (5,20). The disappointing clinical activity was due to their poor selectivity, pharmaceutical properties and toxicity profiles in patients (21,22). Given this precedent, we planned to identify novel Hsp90 inhibitors with a superior pharmacological and tolerability profile. We previously identified a new class of 3,4-isoxazolediamides (17), where the compound SST0116CL1 was selected as a potential new drug candidate, unrelated to the ansamycin class of natural products. We chose to investigate molecules with innovative changes and to assess the benefits of such changes through well-focused *in vivo* studies, based on suitable tumor models.

SST0116CL1 in the studies *in vitro* was shown to inhibit recombinant Hsp90α and to induce the degradation of the oncogenic Her2 tyrosine kinase in BT-474 human breast cancer cells. The degradation of the oncogenic Her2 tyrosine kinase represents a biological effect observed upon addition of known Hsp90 inhibitors to cancer cells (22). Overexpression of Her2 in cancer cells, such as breast and ovarian carcinoma cells, usually results in Akt activation which in turn promotes cell survival. Hsp90 inhibitors induce Her2 degradation through disruption of the Her2/Hsp90 association, and this effect is detrimental to the cell leading to its death (23). Moreover, SST0116CL1 was able to induce the destabilization and depletion of different client proteins, often overexpressed and constitutively activated in numerous types of hematological or solid human tumors. These results well correlated with those obtained from the cell proliferation assay as well as with the Hsp90 competitive binding assay, and clearly confirmed that target modulation of Hsp90 was achieved. We also showed a putative broad spectrum of antiproliferative activity on a panel of tumor cell lines harboring different gene mutations: K-Ras mutations (H460), EGFR and Her2 amplification (A431 and BT474, respectively), N-Ras mutation (HT1080), PTEN loss (A2780), FLT3 mutation (MV4;11) and c-met amplification (GTL-16).

Although the *in vitro* activity of SST0116CL1 was not particularly potent with respect to analogues Hsp90 inhibitors currently undergoing clinical trials, it showed, however, a great versatility and a good pharmacological profile when assessed *in vivo* for tolerability, pharmacokinetics, pharmacodynamics and antitumor activity. In some solid and haematological tumor xenograft models, SST0116CL1 delivered intravenously or intraperitoneally at different schedules, from once a day to once every two or four days (qdx5/w; q2d/w; q4d/w), in both sensitive and doxorubicin-resistant tumor models, showed a statistically significant tumor growth inhibition with a TVI ranging from 61 to 90%, associated to a slight body weight loss (BWL) during the drug-treatment. Three tumor cell lines were selected as tumor xenograft models to represent a diversity of tumor types and oncogenic drivers. The GTL-16 tumor cell line was chosen because of amplification of the receptor tyrosine kinase c-Met, a client protein of Hsp90, and for its dependency on c-Met for growth and survival (24), MV4; 11 leukemia driven by the tyrosine kinase receptor FLT3ITD mutation, was also analyzed. The activating internal tandem duplications (ITD) in the juxtamembrane domain of FLT3 have been identified in 35% AML patients (25). MV4;11 has been shown to be dependent on FLT3-ITD by its sensitivity to selective FLT3 kinase inhibitors (26). The best approach to the treatment of FLT3-ITD AML is currently undefined and multiple clinical trials are investigating inhibitors of the FLT3 kinase (27). Their action is very often transient, possibly due to inadequate dosing or insufficient selectivity of these drugs. SST0116CL1 treatment of the MV4;11 AML resulted in eradication of a good percentage of tumors after subcutaneous tumor cell implantation.

Since many types of cancer express relatively high levels of P-glycoprotein, a major type of MDR protein (28), we show that, unlike Hsp90 inhibitors such as 17-AAG and its derivatives (29), SST0116CL1 exhibited no MDR dependency in A2780/ADR tumor xenograft model. This characteristic makes SST0116CL1 a potentially superior Hsp90 inhibitor in the situation where P-gp is expressed, enabling it, to overcome the MDR barrier that commonly undermines cancer therapy. Differently, 17-AAG could itself induce P-gp expression, rendering the drug less effective during treatment (29).

A modulation of PD biomarkers in terms of downregulation of EGFR, AKT and CDK4 client proteins was achieved either *in vitro*, on A431 tumor cells treated with SST0116CL1, and in terms of down-modulation of c-Met, AKT and CDK4 *ex vivo* in tumor lesions collected from GTL-16 tumor-bearing mice. The molecular signature of Hsp90 inhibition represents a fundamental pharmacodynamic biomarker of efficacy in cancer cell lines, and has been well validated in human tumor xenografts as well as to measure target inhibition in cancer patients receiving treatments with selective Hsp90 inhibitors (30–32). It has been extensively demonstrated that targeting cellular Hsp90 protein function with pharmacological doses of Hsp90 inhibitors results in the depletion of the well-established client proteins, mainly represented by oncogenic proteins or, more generally, cellular proteins involved in the modulation of critical cell pathways and mechanisms, such as cell cycle, proliferation and survival, and in the upregulation of other members of the Hsp90 family. An advantage of Hsp90 inhibitors is their ability to affect multiple oncoproteins simultaneously. This is relevant given the emerging data showing resistant phenotypes arising from mutation, activation of alternative signaling pathways or feedback loops, frequently seen with therapeutics targeting a single oncogene or pathway (33). The preclinical data of tolerability, manageability and activity profile make SST0116CL1 a very attractive antitumor therapeutic agent ready to undergo clinical studies.

## Figures and Tables

**Table I:** Binding affinity to recombinant human HSP90α and Her2 degradation on BT474 breast carcinoma cells.

Compound	HSP90α (IC_50_ ± SD, μM)	Her2 (IC_50_ ± SD, μM)
SST0116CL1	0.21±0.03	0.20±0.02

Competitive binding fluorescence polarization assay and Her2 AlphaLISA Immunoassay were used as described in Materials and methods. IC_50_ values were the average of at least three independent determinations.

**Table II:** Antiproliferative activity of SST0116CL1 on different tumor cell lines.

Cell line	Tissue	SST0116CL1 IC_50_ ± SD (μM)	Alterated oncogenic pathway
A431	Epidermoid carcinoma	0.81±0.02	EGFR
NCI-H460	NSCLC	0.11±0.1	KRAS
A2780	Ovarian carcinoma	0.81±0.1	PTEN
MV4;11	Acute monocytic leukemia	0.40±0.07	FLT3
GTL-16	Gastric carcinoma	0.23±0.04	MET
BT474	Breast carcinoma	0.62±0.1	HER2
HT-1080	Fibrosarcoma	0.34±0.09	NRAS

Tumor cells were treated for 72 h with different concentrations of SST0116CL1 as described in Materials and methods. Results are expressed as average IC_50_ values (± SD) for three independent experiments.

**Table III:** Model independent pharmacokinetic analysis of SST0116CL1 in the plasma and tissues of tumor-bearing mice (A431 epidermoid carcinoma).

Tissue	T_max_ (h)	C_max_ (ng/ml)	AUC_last_ (h*ng/ml)	HL_Lambda_z (h)	CL_F_obs (ml/h/kg)	AUCinf_obs (h*ng/ml)	Vz_F_obs (ml/kg)
Plasma	1.00	2,953	6,822	5.8	11,268	7,100	94,551
Lung	1.00	4,890	21,676	6.5		23,287	
Tumor	2.00	4,046	54,387	NE		267,987	

AUC, area under the concentration vs. time curve.
